# Precipitating hydrophobic injectable liquid (PHIL) embolic for the treatment of a uterine arteriovenous malformation: a technical report

**DOI:** 10.1186/s42155-019-0059-z

**Published:** 2019-05-17

**Authors:** Dylan Kurda, Geetha Guduguntla, Julian Maingard, Hong Kuan Kok, Shivendra Lalloo

**Affiliations:** 1 Interventional Radiology, The Canberra Hospital, Canberra, Australia; 2 Interventional Radiology, Austin Health, Melbourne, VIC Australia; 3 School of Medicine, Deakin University, Waurn Ponds, Geelong, Australia; 4 Interventional Radiology, Northern Health, Melbourne, VIC Australia

**Keywords:** Embolization, PHIL, Uterine artery, AVM

## Abstract

**Objective:**

Uterine arteriovenous malformations (AVM) are unusual causes of vaginal bleeding. Although hysterectomy is the definitive treatment; uterine artery embolization (UAE) provides an alternative therapeutic option. This case presents a technical report of a uterine AVM treated successfully with transcatheter UAE using precipitating hydrophobic injectable liquid (PHIL) embolic agent.

**Case report:**

A 41-year-old female, gravida 6, para 4, miscarriage 2, including a molar pregnancy 15 years prior, presented with massive per vaginal bleeding. Pelvic ultrasound demonstrated an acquired AVM as the underlying aetiology for her presentation. The patient underwent bilateral uterine arterial embolization. Four weeks later, there was nearly complete resolution of the AVM and the patient’s menstrual cycle was restored 8 weeks after the procedure.

**Conclusion:**

Uterine AVM can be treated safely and effectively with UAE using PHIL.

## Introduction

Uterine arteriovenous malformation (AVM) is a rare cause of menorrhagia that may cause life threatening bleeding. The diagnosis is most commonly established by pelvic ultrasound although other cross-sectional imaging modalities including CT and MRI are also helpful. Uterine artery embolization (UAE) is one of the therapeutic options, for which several embolic agents have been mentioned in literature. The first successful transarterial embolization for uncontrolled uterine bleeding from fibroids was performed in 1974 by Jean-Jaques Merland (Yoon et al., 2016; Sellers et al., 2013) and UAE is now an established first-line treatment option for symptomatic fibroids.

Although there is more limited experience with UAE for treatment of uterine AVM, the choice of technique and embolic agent can impact on the success of endovascular embolization with varying rates of primary occlusion and recurrence reported in the literature^1^. We report our preliminary experience using a novel liquid embolic agent, precipitating hydrophobic injectable liquid (PHIL; MicroVention, Tustin, CA) demonstrating safety and efficacy for the treatment of a large post-partum uterine AVM.

## Materials and methods

A 41-year-old gravida 6, para 4 (G6P4) was referred to our institution following large volume per vaginal (PV) bleeding with an estimated 900 ml blood loss. Her past history was significant for two miscarriages, one resultant from a molar pregnancy 15 years prior. She last gave birth to a normal term baby 4 months prior to this presentation; which was a normal vaginal delivery. Her immediate postpartum course was uneventful, without complications such as sepsis and retained product of conception. She was on a regular dose of the combined oral contraceptive pill.

**Fig. 1:**
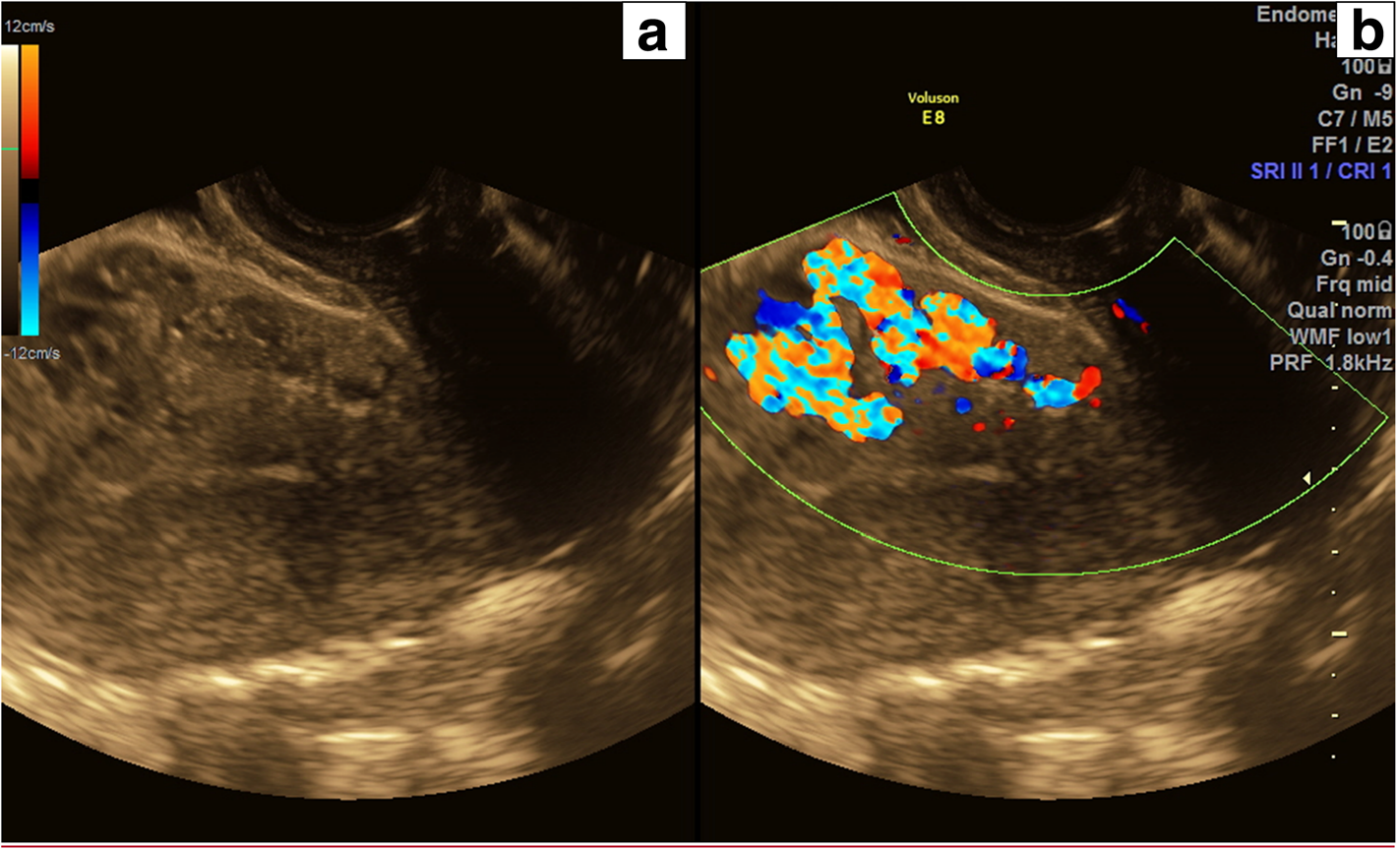
**a** Sagittal transvaginal pelvic ultrasound image demonstrating a serpinginous hypoechoic area in the anterior myometrium compatible with an anterior cornual AVM. **b** Colour Doppler overlay demonstrating markedly increased high-flow vascularity of the AVM

Upon this presentation, she was initially treated with 2 units of packed red cells, tranexamic acid, norethisterone and naproxen but her symptoms failed to respond to medical therapy. Duplex pelvic ultrasound demonstrated an anteverted uterus with changes of adenomyosis and a left cornual AVM measuring 4x3x3 cm in the anterior myometrium (Fig. 1a and b). She was referred for transcatheter UAE to treat the underlying AVM.

## Procedure details

**Fig. 2:**
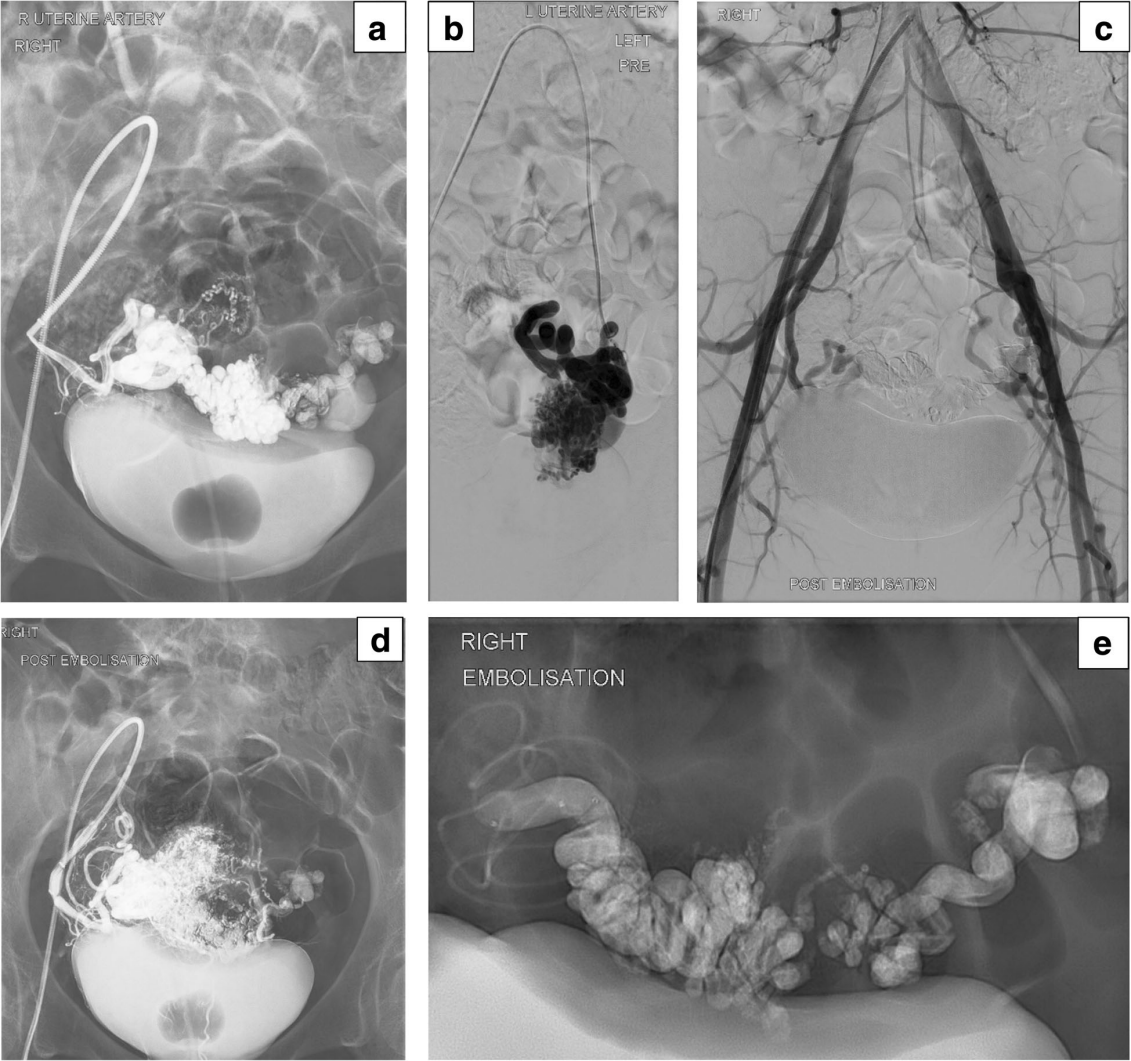
**a-e.** 41-year-old female with symptomatic acquired uterine AVM. **a** Right uterine artery demonstrates hypertrophied right uterine artery and AV shunting, with draining venous varix; **b** Left uterine artery with evidence of AV shunting and a well demonstrated nidus; **c** Aortogram post bilateral UAE, showing obliteration of the AV shunting and no further feeder from internal and / or external iliac arteries; **d** Post right uterine artery embolisation with PHIL, showing no evidence of AV shunting. Adequate nidus penetration with the embolic cast also seen; **e** Post bilateral UAE demonstrating PHIL cast and the Scepter XC catheter. It also nicely demonstrates the marked tortuosity of the hypertrophied right UA

Following informed consent and under conscious sedation, retrograde right common femoral artery access was obtained followed by placement of a 6-French introducer sheath (Cook, Bloomington, IN). The left and right internal iliac arteries were selectively catheterised using a 5-French C2 catheter (Glidecath, Terumo) and hydrophilic guidewire combination (Glidewire, Terumo). Selective angiography of the internal iliac artery demonstrated an anterior left paramedian uterine AVM nidus supplied by both uterine arteries and an associated post-nidal varix. We used 5000 units of intraarterial unfractionated heparin (we normally do use heparin in such situations due to potential lengthy procedures and the use of long sheaths / guide catheters). Distal catheterization of the markedly tortuous uterine arteries was performed using a dual lumen Scepter XC balloon microcatheter and Traxcess wire combination (Microvention) delivered through a 6 Fr guide sheath (Destination,Terumo) for proximal support. Pulsed injection of 7 ml of 25% and 2 ml of 30% PHIL liquid embolic (to bilateral uterine arteries) achieved satisfactory nidal penetration, with post embolization aorto-iliac angiography suggesting no further early venous filling. Despite the lack of post-nidal venous penetration which may allow recurrent shunting, adequate devascularization was achieved (Fig. 2 a-e). Access site hemostasis was achieved with a vascular closure device (AngioSeal, Terumo).

## Results

**Fig. 3:**
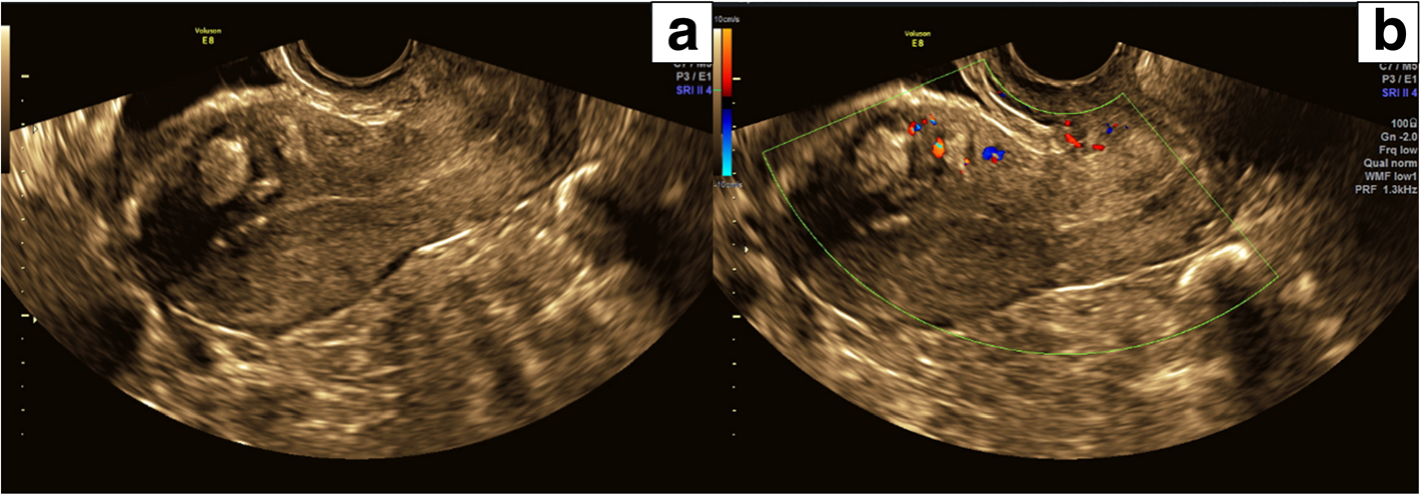
**a** Sagittal transvaginal pelvic ultrasound image 2 weeks post UAE demonstrating echogenic PHIL embolic material in the anterior myometrium at the site of the treated AVM **b** Colour Doppler overlay demonstrating markedly reduced vascularity

The patient recovered well; her PV bleeding tapered off over a 10-day period and subsequently ceased. Follow-up pelvic ultrasound demonstrated obliteration of the nidus with no residual vascularity within the region of the original AVM (Fig. 3). She restarted her oral contraceptive pill post-embolisation. Her post-embolisation course was uneventful apart from mild pelvic pain in the first few days post procedure which was adequately managed with oral analgesics. There was no clinical evidence of post embolization syndrome. The patient remained well at 5 month follow-up and was asymptomatic.

## Discussion

Uterine AVMs are rare, and their true incidence is not clear. They are believed to be underestimated as they are often clinically silent prior to late presentation with severe PV haemorrhage (Sellers et al., 2013). Approximately 50% of uterine AVMs are congenital and thought to be the result of failure of embryonic capillary plexus differentiation (Kasznica & Nisar, 1995). The remainder of AVMs are acquired after miscarriage, uterine instrumentation, Caesarean section or other uterine surgery, or related to neoplastic disorders like gestational trophoblastic disease and endometrial adenocarcinoma. Maternal diethylstilboestrol (DES) use has also been implicated.

In the puerperium, there is a recognized continuum from self-limiting subinvolution of retained placental vasculature to persistent pathologic shunts which present as symptomatic acquired AVMs (Sellers et al., 2013). First-line imaging includes assessment with pelvic duplex ultrasound; although, cross sectional imaging can help to characterise the exact nature and relationship to surrounding structures including the number and location arterial in-feeders, location and size of the nidus as well as the presence of venous varices and flow related aneurysms (Timor-Tritsch et al., 2016; Kwon & Kim, 2002). Cross sectional imaging has the added benefit of allowing accurate characterisation of residual nidus following treatment.

Management of uterine AVMs is dictated by the clinical status and age of the patient, site and size of the AVM, and the desire for gestation in the future (Molvi et al., 2011). Percutaneous transarterial embolization has become established as an effective treatment for uterine AVMs with lower morbidity and mortality, over the surgical alternatives of vessel ligation or hysterectomy (Ghai et al., 2003; Peitsidis et al., 2011; Touhami et al., 2014). Advantages of embolization include prompt and accurate identification of bleeding site, preservation of uterus and subsequent fertility as well as reduced recurrence of bleeding from collaterals due to ability to perform more distal occlusion of the nidus. However despite its widespread use, a recent systematic review concluded that only low-level evidence supported the role of embolization and in light of the heterogeneity of pathology, embolic agents and approaches, it emphasized the importance of refining the procedural protocol (Yoon et al., 2016).

Regardless of its location, the goal of AVM embolization is complete obliteration of the nidus, while avoiding unwanted non-target reflux into the feeding arteries or premature embolization of its draining veins. In situations where tissue or end-organ death is a desired end point, permanent embolic agents and smaller particles are the preferred choice (Koçer et al., 2016a). Vascular abnormalities, including AVMs, can be successfully treated by permanent occlusion as the host organ can remain well supplied by collaterals (Lubarsky et al., 2010).

PHIL, a novel liquid embolic agent, is a ready-to-use non-adhesive copolymer. It is dissolved in dimethyl sulfoxide (DMSO), and does not require any preparation and is covalently bound to its iodine component, which provides its inherent radiopacity (Vollherbst et al., 2017). PHIL liquid embolic system consists of a sterile, prefilled 1.0 mL syringe of the liquid embolic agent, a sterile, prefilled 1.0 mL syringe of DMSO, and microcatheter hub adaptors. A DMSO-compatible delivery microcatheter that is indicated for use in the neurovascular or peripheral vasculature is used to access the target for embolization. PHIL is delivered by slow, controlled injection under fluoroscopic control. The DMSO solvent dissipates into the blood, causing the copolymer to precipitate forming an embolus. PHIL polymerizes from the outside to the inside, while penetrating distally in the vascular lesion. Final solidification of the injected agent occurs within 5 min according to the manufacturer (Leyon et al., 2016).

The non-adhesive nature of this agent affords potential for longer injection times as well as the capacity to perform control angiography mid-procedure. Its preference as an embolic agent in the management of cerebral AVMs and dural arteriovenous fistulae is increasing (Koçer et al., 2016a; Samaniego et al., 2016; Lamin et al., 2017).

The use of other liquid embolics, for example N-butyl cyanoacrylate (NBCA) and ethylene vinyl alcohol copolymer (Onyx), in the management of uterine AVMs has been reported previously with favourable clinical results. Compared to Onyx, shorter intervals (30–60 s) are required during injection of PHIL (2 min in the case of Onyx), which allows a greater margin for reflux (2 cm). There is less streak artefact with PHIL compared to Onyx on follow-up CT imaging. The PHIL agent does not contain micronized tantalum unlike Onyx; therefore, PHIL is more homogenous and does not require prior agitation prior to use (Hemingway, 1984). The radiation dose is perhaps smaller for the patient and the operator in case of PHIL due to a quicker injection time and the ability to change the radiation factors for visualization during the procedure (Hemingway, 1984), which is of added value for pelvic angiographic procedures in young females. However, PHIL has limited visibility during passage through the microcatheter (especially PHIL 25%). After passage through the tip of microcatheter, the visibility of PHIL is lower than Onyx (Koçer et al., 2016a).

Gel foam is a biological substance prepared from purified skin gelatin. Related studies mostly support preservation of menstrual function and fertility following uterine embolization with gel foam, with one study reporting resumption of menses in all of their patients. Another study reported 98% preservation of menses and 82% successful future pregnancies (Touhami et al., 2014; Farouk et al., 2016; Cheng et al., 2017).

Despite literature supporting preservation of fertility, gel foam poses some inherent disadvantages as an embolic agent for uterine AVM embolization compared to newer agents. As a temporary embolic agent, recanalization can occur in the weeks following embolisation (Lubarsky et al., 2009). The smaller size of the particles in gel foam powder preparation, also results in increased risk of potential and unwanted ischemia from non-target reflux (Siskin et al., 2000).

Our patient did not experience any post-embolisation complication, including major pain, infection and post-embolisation syndrome (PES). PES is the most common complication following UAE for uterine AVMs (Hughes & Reidy, 2003). One study looking at a large variation in indication for embolisation, both in location and pathology, demonstrated a lower incidence in PES when treatment was for acute bleeding (Hemingway, 1984). It was inferred that this was in relation to decreased volume in of necrotic tissue or thrombosed vasculature, as point of occlusion is as directed as possible (Hemingway, 1984). A case series demonstrated that, unlike its counterpart liquid embolic agent Onyx, PHIL causes moderate vascular inflammatory effect but no angionecrosis (Koçer et al., 2016b). This might be related to possible reduced PES rate after PHIL.

## Conclusion

In conclusion, we report a case of successful endovascular treatment of a uterine AVM with the novel liquid embolic agent PHIL. Further studies are needed to establish possible superiority and fertility preservation over other embolic agents such as Onyx.

## Abbreviations

AVM: Arterio venous malformation
PHIL: Precipitating hydrophobic injectable liquid
UAE: Uterine artery embolization
